# An equilibrium desorption model for the strength and extraction yield of full immersion brewed coffee

**DOI:** 10.1038/s41598-021-85787-1

**Published:** 2021-03-25

**Authors:** Jiexin Liang, Ka Chun Chan, William D. Ristenpart

**Affiliations:** 1 Department of Food Science and Technology, University of California, Davis, One Shields Avenue, Davis, CA 95616 USA; 2 Department of Chemical Engineering, University of California, Davis, One Shields Avenue, Davis, CA 95616 USA

**Keywords:** Chemical engineering, Chemistry

## Abstract

The sensory qualities of brewed coffee are known to be strongly correlated with the total dissolved solids (TDS) and extraction yield (*E*) of the brew. Here, we derive a predictive model for the TDS and *E* of full immersion brewed coffee using a pseudo-equilibrium desorption approach. Assuming a single, species-averaged equilibrium constant $K$ yields theoretical predictions indicating that the TDS is approximately inversely proportional to the water/coffee mass brew ratio, while *E* is independent of the brew ratio. Our experimental results strongly accord with both theoretical predictions, and indicate that *E* is approximately 21% over a wide range of brew ratios. An analysis of the standard oven-drying method for measuring *E* indicates that it yields significant underestimates of the true value at equilibrium, due to retained brew within the spent moist grounds. We further demonstrate that $K$ is insensitive to grind size, roast level, and brew temperature over the range 80–99 °C. Taken together, our results indicate that full immersion brewing offers precise control over the TDS at equilibrium but little control over *E*, and that practitioners should pay careful attention to their brew ratio as the most important parameter for full-immersion brewing.

## Introduction

The immense popularity of coffee beverage is driven in part by its complex sensory profile^1–3^, which in turn is due to its complicated chemical composition: brewed coffee contains several hundred different species that are known to affect the sensory profile^4^, the concentrations of which can be modified by choice of brewing technique^5,6^. Because of this complexity, many investigators and coffee industry practitioners focus not on the specific chemical composition, but instead quantify the mass concentration of all dissolved species in the beverage. This quantity, also known as the “brew strength,” is often characterized as the “total dissolved solids” (TDS), and can be related by mass conservation arguments^7^ to the extraction yield *E* from the solid coffee grounds (sometimes also known as the percent extraction, PE). Early work by Lockhart established that the TDS and *E* are good indicators for the quality of coffee, with TDS values near 1.25% and *E* values near 20% identified as “ideal.”^8^ These early results have more recently been updated and expanded with modern sensory methodologies to better capture the broad range of flavors possible in brewed coffee^9,10^. In particular, systematic experiments showed that higher TDS is strongly associated with increased bitterness, smoky, and roast-like aromas; higher TDS and low *E* is associated with higher sourness and citrus; while lower TDS is associated with increased sweetness and tea-like/floral at low or high *E* respectively. These differences in sensory attributes help drive consumer preference of the same roasted coffee brewed to different strengths and extraction yields^11^.

Given the importance of TDS and *E* in the sensory profile of brewed coffee, a natural question is: how do we predict these quantities in terms of brewing control parameters, like the brew ratio, brewing temperature, water flowrate, and coffee grind size? Towards that end, several groups have examined theoretical models aimed at predicting the brew strength and extraction yield. Fasano and Talamucci presented mathematical models primarily focused on describing the mass transport phenomena taking place in an espresso machine^12^. Moroney and colleagues used multiscale methods to characterize the spatial–temporal dynamics of coffee extraction by hot water from a fixed coffee bed^13^, and subsequently developed a model reduction of this work to describe coffee extraction kinetics in a well-mixed system^14^. They further compared one-dimensional flow models to computational fluid dynamics modelling for packed bed coffee extraction^15^. Melrose et al. introduced a simpler “base model” for coffee brewing through a packed bed of coffee grains to generate TDS and extraction yields versus brew parameters^16^. Most recently, Cameron et al. developed a detailed model for espresso extraction with the assumption of homogeneous flow through the coffee bed, yielding predictions for how to increase the extraction yield^17^.

Notably, almost all of the above theoretical work focused on flow extractions, where fresh water flows into the grounds and then extracted brew flows out the other side. Another equally important class of brewing technique, however, involves “full immersion” brewing. Also known as “steeping,” this method involves immediate addition of all of the water to the coffee grounds, and then waiting for the extraction to proceed before finally removing the coffee grounds. A classic example is the well-known “French press” style of brewer, but other brewer types such as Toddy-style cold brewers or Japanese vacuum brews also involve full immersion. A particularly important type of full immersion brewing is “cupping,” which is the traditional method that coffee industry professionals around the world use to assess the quality of different coffee lots^18,19^. Cupping traditionally involves full immersion of a rigidly prescribed amount of hot water to coffee grounds, with no filtration except skimming off floating coffee grounds with a spoon at a certain time point following the addition of the hot water^20^.

Despite the widespread popularity of full immersion brewing methods, relatively little theoretical work has examined it. Moroney et al. used their multiscale flow extraction model to investigate the limiting case of batch-wise brewing^13,14^; they solved the resulting set of coupled differential equations numerically^13^ and via matched asymptotic expansions^14^ to assess TDS versus time. This approach, however, required multiple fitting parameters, and to date several key questions about full immersion brewing remain unanswered. For example, how does the brew ratio affect the strength of full-immersion coffee? How does it affect the extraction yield? What is the impact of the brew temperature, grind size, and roast level on these quantities?

Here, we develop a theoretical model for full-immersion brewing of coffee to answer these questions. We assume the beverage solution and solid coffee grounds reach an effective equilibrium between desorption and adsorption that can be approximated with a single equilibrium constant reflecting the average behavior of all chemical species present. The resulting model predicts that the brew strength varies approximately inversely with brew ratio, but that the extraction yield at equilibrium is independent of the brew ratio. The model further predicts that retained liquid following filtration will yield artificially low measurements for the extraction measured via the standard oven drying approach. Experimental measurements accord with these model predictions, and further indicate that the effective equilibrium constant is insensitive to roast level, grind size, and brew temperature at least over the range of 80 to 99 °C. The results presented here provide insight on how full immersion brewing techniques can be optimized to yield desired brew strength that may yield desired sensory profiles^11^.

## Theory

### Definition sketch for full immersion brewing

**Figure 1:**
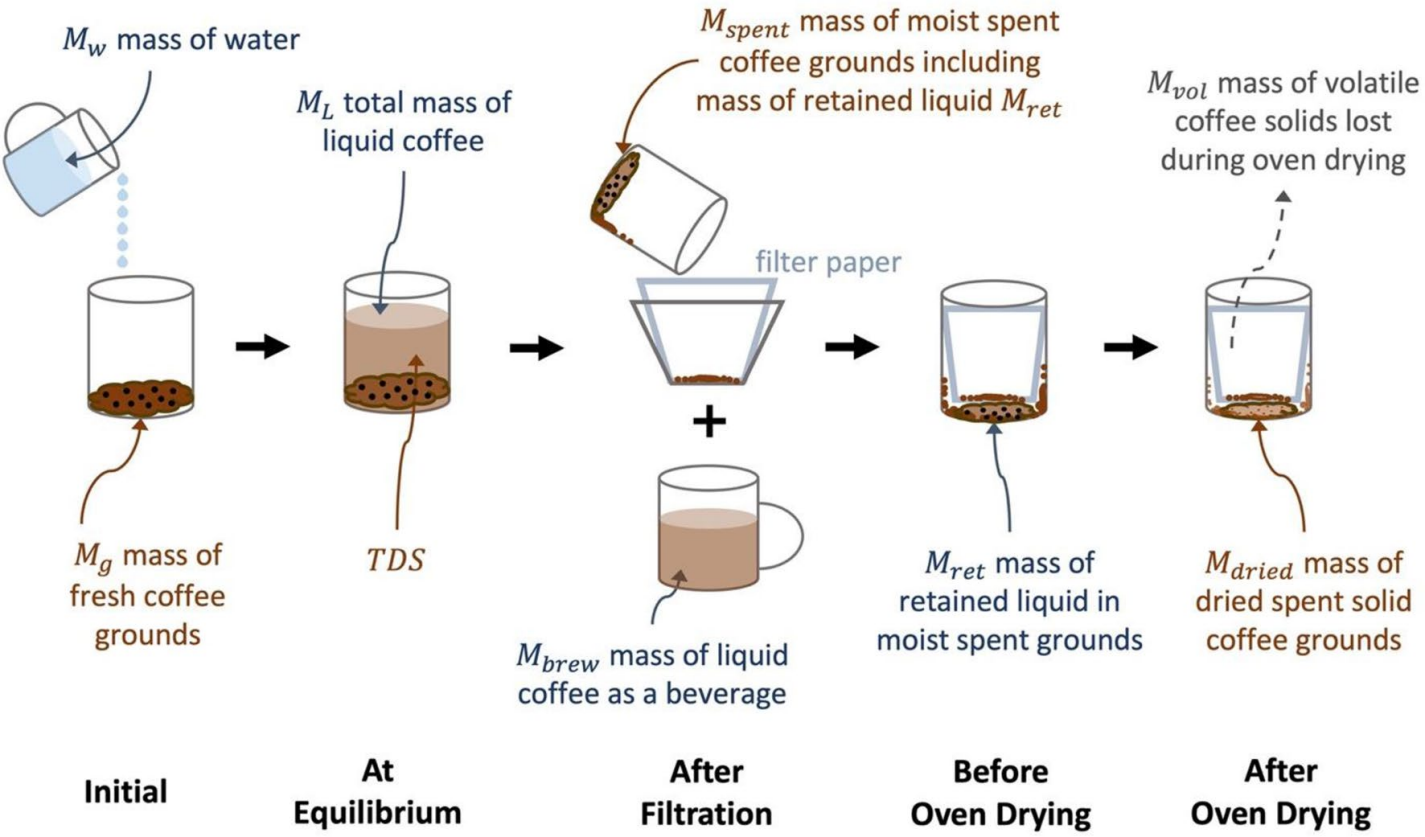
Schematic of a full immersion brew with subsequent oven-drying measurement of extraction.

The main goal of this section is to derive theoretical predictions for the TDS and *E* of full immersion brew, focusing on the brewing and characterization process depicted schematically in Fig. 1 and using nomenclature summarized in Table S1. Our approach is based on well-established principles in chemical engineering^21^, but which to our knowledge have not been used in the context of coffee. Initially, a known mass of coffee grounds (${M}_{g}$) and a known mass of hot water (${M}_{w}$) is added to the brewer. The ratio of these masses is defined as the brew ratio,

1 $$R_{brew} = \frac{{M_{w} }}{{M_{g} }}.$$

Upon addition of the water to the coffee grounds, mass transfer proceeds and the TDS of the liquid increases and eventually plateaus to a steady value (behavior that is readily verified experimentally, as discussed below). We neglect the potentially complicated dynamics that occur during the transient phase to focus on the final ‘equilibrium’ brew strength of the liquid. This condition is not a true thermodynamic equilibrium since the brewed coffee can continue to undergo subtle changes (such as evaporation of volatiles or gradual acidification due to slow chemical reactions), but for the time scales of interest (measured in minutes or tens of minutes) we assume the system achieves an effective pseudo-equilibrium.

After the brew strength reaches a final steady value, the brew is then filtered, yielding a mass of beverage ${M}_{brew}$ that could be consumed, and leaving behind a mass ${M}_{spent}$ of spent moist coffee grounds. Finally, a standard procedure to measure the overall extraction of the brew is to place the spent moist grounds in an oven to bake off the water and measure the residual mass. As discussed in detail below, ${M}_{spent}$ contains both the unextracted coffee grounds plus some retained beverage that contains part of the dissolved coffee species, complicating interpretation of the measured extraction yield via oven drying.

### Equilibrium desorption model

We focus first on a prediction for the equilibrium TDS. Ground coffee contains hundreds of individual chemical species, all of which have a unique solubility: some species are highly soluble and will completely dissolve, while others are weakly soluble and will hardly dissolve. Instead of tracking individual species, we assume that the overall behavior can be described by a single, average equilibrium constant with corresponding rates of desorption and adsorption from and onto the solid insoluble coffee ground matrix. Specifically, these rates are related as

2 $${C}_{A} \begin{array}{c}{k}_{D}\\ \rightleftharpoons \\ {k}_{A}\end{array} {C}_{D},$$

where ${C}_{A}$ is the mass concentration of adsorbed soluble coffee species, ${C}_{D}$ is the mass concentration of dissolved soluble coffee species, ${k}_{D}$ is the first order rate constant of desorption, and ${k}_{A}$ is the first order rate constant of adsorption. For simplicity in relating theory to experimental observations, both mass concentrations are per unit mass of liquid, ${M}_{L}$, such that

3a $$C_{A} = \frac{{M_{A} }}{{M_{L} }},$$

3b $$C_{D} = \frac{{M_{D} }}{{M_{L} }}.$$

By this definition, ${C}_{D}$ is equivalent to the TDS. We assume here that volatilization of the coffee solids is negligible, so the soluble coffee species are either dissolved in solution in liquid phase, or adsorbed on the solid coffee grounds. Accordingly, the total mass of soluble species is conserved,

4 $${M}_{A}+{M}_{D}={M}_{tot}$$

where ${M}_{tot}$ is the total mass of extractable coffee species (and ${C}_{tot}={M}_{tot}/{M}_{L}$ is the total concentration). This quantity can be expressed in terms of the maximum extraction yield possible in the coffee,

5 $${M}_{tot}={E}_{max}{M}_{g},$$

where ${E}_{max}$ is the maximum extraction of coffee grounds. Typically ${E}_{max}$ is observed to be around 0.3, that is, only about one third of the mass of roasted coffee is soluble in water^13^.

After the initial complicated dynamics that occur when the water is added to the coffee grounds, the brew eventually reaches equilibrium such that the concentrations reach a steady state,

6 $$\frac{d{C}_{A}}{dt}=0=-{k}_{D}{C}_{A}+{k}_{A}{C}_{D},$$

which with Eq. (4) yields

7 $$C_{D} = \left( {\frac{{k_{D} }}{{k_{D} + k_{A} }}} \right)C_{tot} .$$

We define the equilibrium constant,

8 $$K=\left(\frac{{k}_{D}}{{k}_{D}+{k}_{A}}\right),$$

and substitution of Eq. (5) yields

9 $${C}_{D}=K{E}_{max}\frac{{M}_{g}}{{M}_{L}}.$$

Note that ${M}_{L}$ is not known a priori but it is readily related to the mass of water added and the mass of dissolved coffee species present in the brew,

10 $${M}_{L}={M}_{w}+{M}_{d}.$$

This formulation assumes that any water mass lost to evaporation, or water mass lost to physical adsorption onto the solid coffee grounds, is negligible. Substitution of (10) into (9), recalling Eq. (1), and rearrangement yields the equilibrium TDS,

11 $${C}_{D}=TDS=\frac{K{E}_{max}}{{R}_{brew}+K{E}_{max}}.$$

Under typical conditions, ${R}_{brew}$ is on the order of 5 to 25, while ${E}_{max}$ is approximately 0.3. Thus, if $K$ is order one or smaller, then to good approximation the second term in the denominator is negligible and we have the simplified expression

12 $$TDS=\frac{K{E}_{max}}{{R}_{brew}}.$$

### Extraction yield at equilibrium

Given the preceding analysis, the corresponding extraction yield at equilibrium is readily derived. By definition, the extraction yield *E* is the mass of solid coffee that dissolves into the liquid phase per original mass of coffee grounds,

13 $$E =\frac{{M}_{d}}{{M}_{g}}.$$

By Eqs. (3b) and (9), we have

14 $${M}_{d} ={C}_{D}{M}_{L}=K{E}_{max}{M}_{g},$$

and substitution back into Eq. (13) yields the final result,

15 $$E =K{E}_{max}.$$

We emphasize that Eq. (15) is the theoretical prediction for the actual extraction yield of the brew at equilibrium, not after drying spent grounds in the oven. Notably, $E$ is predicted to be completely independent of the brew ratio, and to depend only on the equilibrium constant and the maximum extraction. In other words, the brewer can arbitrarily vary the strength of the brew as per Eq. (12) simply by altering the brew ratio, but the extraction yield at equilibrium is adjustable only by altering the ingredients: in principle, both $K$ and ${E}_{max}$ could depend on the composition of the coffee, the composition of the water, and the system temperature.

A key point is that the extraction yield at equilibrium cannot be measured directly, but we can relate it to the TDS using mass conservation arguments. Specifically, from Eqs. (3b) and (10) the mass of dissolved solids is related to the TDS as,

16 $${M}_{d} =\frac{TDS}{1-TDS}{M}_{w},$$

and substitution into Eq. (13) yields

17 $$E =\frac{TDS}{1-TDS}{R}_{brew}.$$

It is straightforward to verify that substitution of Eq. (11) into Eq. (17) yields $E =K{E}_{max}$, i.e., expressions (15) and (17) are self-consistent. Because the TDS is readily measured experimentally, Eq. (17) provides an easy means to assess the extraction yield of a full-immersion brewed coffee.

### Extraction yield from the oven drying method

Finally, we consider a theoretical prediction for extraction yield when measured experimentally using the oven drying method. In this technique, the brewed liquid is filtered from the spent grounds, and the spent moist coffee grounds are dried in an oven to remove all the water (Fig. 1). The extraction yield can then be calculated using the difference of coffee grounds before brewing and after drying,

18 $${E}_{oven}=\frac{{M}_{g}-{M}_{dried}}{{M}_{g}},$$

where ${M}_{dried}$ is the experimentally measured mass of dried spent grounds. Note that $E\ne {E}_{oven}$ because they measure different quantities: $E$ reflects the amount of dissolved coffee at equilibrium in the beverage, while ${E}_{oven}$ reflects all solids left behind after filtration and baking. In particular, the spent moist grounds invariably include some retained brew with dissolved solids that must be accounted for, and baking the grounds for several hours can cause some fraction of the mass to volatilize and escape into the gas phase. By conservation of mass, the mass of dried grounds must equal the original mass of coffee grounds minus what was removed by brewing and baking,

19 $${M}_{dried}={M}_{g}-TDS\cdot {M}_{brew}-{M}_{vol}$$

Here ${M}_{brew}$ is the mass of brewed coffee delivered into a separate container following filtration, while ${M}_{vol}$ is the mass of coffee solids volatilized while baking in the oven. Note that ${M}_{brew}$ depends on how effectively one pours off the brew (i.e., one can leave behind a little or a lot of liquid amongst the spent grounds), but in any case ${M}_{brew}$ is readily measured. Substitution of Eq. (19) into Eq. (18) yields

20 $${E}_{oven}=TDS\frac{ {M}_{brew}}{{M}_{g}}+\frac{{M}_{vol}}{{M}_{g}}.$$

If we define ${M}_{ret}$ as the mass of liquid retained within the spent moist grounds following filtration, then

21 $${M}_{brew}={M}_{L}-{M}_{ret},$$

and substitution of Eqs. (9) and (15) yields

22 $${E}_{oven}=K{E}_{max}\left(1-\frac{ {R}_{ret}}{{R}_{brew}+K{E}_{max}}\right)+{R}_{vol}.$$

Here ${R}_{ret}={M}_{ret}/{M}_{g}$ is the liquid retention ratio during filtration and ${R}_{vol}={M}_{vol}/{M}_{g}$ is the volatilization ratio during baking. If the latter quantity is negligible, and for brew ratios such that ${R}_{brew}\gg K{E}_{max}$, then to good approximation we have

23 $${E}_{oven}=K{E}_{max}\left(1-\frac{ {R}_{ret}}{{R}_{brew}}\right)$$

In other words, the extraction yield as measured via oven drying will invariably be smaller than the true extraction yield at equilibrium, with the magnitude of the discrepancy decreasing as the brew ratio increases. The retention ratio depends on how efficiently one pours off the brew, but to calculate it, substitution of Eqs. (10) and (16) into Eq. (21) and rearrangement yields

24 $${R}_{ret}=\frac{{R}_{brew}}{1-TDS}-\frac{{M}_{brew}}{{M}_{g}}.$$

All of the terms on the right-hand side of Eq. (24) are readily measured experimentally.

## Experimental methodology

The main goal of the experimental work was to assess the validity of Eqs. (11), (15), and (22) for the equilibrium TDS, $E$, and ${E}_{oven}$ respectively. Two separate suites of experiments were performed. First, a single type of coffee was tested systematically in 1-L full immersion brews, using the methodology presented in Fig. 1, for a wide variety of brew ratios, brew temperatures, and grind sizes. Second, because the traditional cupping method is used so ubiquitously in the coffee industry, we tested five different coffees at different roast levels with the cupping method over a wide range of brew ratios. Specific brewing parameters for each brewing experiment are summarized in Table 1. The study complied with all relevant institutional, national, and international guidelines and legislation.

**Table 1:** Specific values of brewing parameters tested in four different brewing experiments. For the 1-L beaker equilibrium full immersion brews, selected combinations of brewing conditions present in Fig. 3 were tested.

Experiment	Brew temperature (°C)	Brew time (minutes)	Brew ratio	Grind size setting^a^	Distinct roasts	Trial replicates	Total number of brews
Dynamics of extraction in 1-L beaker full immersion brews	80, 94, 99	1, 3, 5, 10, 20, 30, 45, 60, 90	5, 25	5	1	3	18
Dynamics of extraction in 300-mL beaker full immersion brews followed by filtration	94	3, 5, 8, 12, 20, 30	12, 14, 16, 18, 20	5	1	3	90
Equilibrium in 1-L beaker full immersion brews	80, 85, 94, 99	60 (80 °C), 45 (85 °C), 30 (94 °C), 20 (99 °C)	2, 3, 4, 5, 6, 10, 13, 15, 20, 17, 25	2, 3, 4, 5, 6 (all at ${R}_{brew}=15$)	1	3	99
Traditional cupping in 12-oz cup full immersion brews	99	14	12 to 21	10	5	3	90

^a^All grind size settings refer to Mahlkönig Guatemala Lab Grinder (cf. Supplementary Fig. S1), except for the traditional cupping which was performed on a Mahlkönig Guatemala 710 Grinder.

### Coffee

For our systematic tests using 1-L full immersion brews (“Dynamics of extraction in 1-L beaker full immersion brews” and “Equilibrium in 1-L beaker full immersion brews”), we used a blend of four African-origin wash-processed Arabica coffees (Rwanda Kivu Kanzu, Rwanda Kivu Kibuye, Rwanda Musasa Mbilima Dukunde, and Ethiopia Yirgacheffe Kebele Kochore) procured from Royal Coffee in Oakland, CA. Each green coffee was roasted separately, and all were roasted at the UC Davis Coffee Center using a Loring demo roaster (Loring Smart Roast, Inc., Santa Rosa, CA, USA) within a 5-day period. The roast levels were 67.2, 70.0, 70.1, and 70.4 respectively, determined by the Agtron Gourmet Color Scale published by the SCA^22^. The coffee was allowed to rest for 2 days after roasting to allow for degassing, and then the four coffees were well-mixed together by hand, vacuum-sealed in 1-kg bags, and stored in a − 20 °C freezer. Each individual bag of coffee was defrosted overnight to room temperature before use.

For our dynamic tests with 300-mililiter brews (“Dynamics of extraction in 300-mL beaker full immersion brews followed by filtration”), we used a single origin wash-processed Arabica coffee from Rwanda Kivu Kanzu provided by Royal Coffee in Oakland, CA. Coffee was roasted to an Agtron Gourmet reading of 70.1 at the UC Davis Coffee Center using a Loring demo roaster on a single day, followed by a 2-day resting period. Then, coffee was well-mixed, vacuum-sealed in 1-kg bags, and stored in a − 20 °C freezer; and individual coffee bag was defrosted in room temperature before use.

For our traditional cupping tests (“Traditional cupping in 12-oz cup full immersion brews”), five different commercially available blends of Arabica coffee were provided by Peet’s Coffee (Alameda, CA, USA). Specifically, we tested Colombia Luminosa, Big Bang, Major Dickason’s Blend, and French Roast, which respectively represent light roast, medium roast, dark, roast, and extra dark roast; note that a “light” roast at Peet’s Coffee is typically darker than roasts characterized as “light” elsewhere. We also tested Decaf House Blend, a decaffeinated dark roast. Each coffee was roasted at Peet’s Roastery in Alameda, CA using a Probat R-Series Solid Drum Roaster. The corresponding Agtron Commercial Color Scale readings were 54.6, 45.0, 35.1, 26.9, and 35.1, respectively. The coffees were ground and brewed on the same day of roasting, without packaging or a degassing period, to mimic standard quality control procedures.

### Experimental methods

#### Dynamics of extraction in 1-L beaker full immersion brews

Preliminary experiments assessed how much time was required to reach equilibrium. The dynamics of full immersion brewed coffee were studied at two different brew ratios and three different brew temperatures. Coffee was ground immediately prior to each experiment using a Mahlkönig Guatemala Lab Grinder (Mahlkönig USA, Durham, NC, USA) at grind size setting 5 with a median particle size, ${x}_{50}$, of 1160.5 ± 28.8 µm. The particle size distribution (cf. Supplementary Fig. S1) of each grind size setting from 2 to 6 were measured in triplicate using a Sympatec HELOS/RODOS T4.1 laser analyzer (Sympatec GmbH, Clausthal-Zellerfel, Germany) with the Sympatec Vibir vibratory feeder and R7 lens. Nestlé Pure Life Purified Water was used for all brews^23^. The pH of this water was measured to be 7.46 by Mettler Toledo SevenCompact Duo S213 pH/Conductivity Meter (Mettler-Toledo LLC, Columbus, OH, USA). Water was heated to a target temperature by an OXO Adjustable Temperature Pour-Over Kettle (OXO, New York City, NY, USA).

Water extraction temperatures were chosen at 80 °C, the low end of brewing temperatures found acceptable by consumers for hot brewed coffee^24^; 94 °C, within the range of brewing temperature suggested by the Coffee Brewing Handbook published by the Specialty Coffee Association^25^; and 99 °C, as close as possible to boiling. The coffee was brewed using a 1-L glass beaker by adding an appropriate amount of water at 80 °C, 94 °C or 99 °C to a constant amount of 30 g of coffee grounds to yield a desired brew ratio. Water was poured in a circular motion to ensure all the coffee grounds were fully wet. The timing of the brews started once water pouring was completed. The beaker sat on a room temperature wooden countertop with no lid; no additional insulation was provided to mimic typical brewing conditions. Accordingly, the temperature decreased after addition of the hot water to the room temperature grounds (cf. Supplementary Fig. S2). At 1, 3, 5, 10, 20, 30, 45, 60 min for all brews, and 90 min for the ${R}_{brew}=5$ brews, since it took longer for the brews at lower brew ratios to reach equilibrium. A single drop of coffee was pipetted out from the brew to measure the TDS at that time points stated above. Aside from this gentle sampling, no stirring was performed. All measurements were performed with three separate trial replicates.

#### Dynamics of extraction in 300-mL beaker full immersion brews followed by filtration

Because the sampling could theoretically affect the extraction dynamics, we also performed tests where a 300-mL brew sat completely undisturbed until a specified brew time, at which point it was filtered through paper and then its TDS measured. The same coffee grinding procedure was used as with the 1-L beaker full immersion brews. Deionized water was heated using an OXO Adjustable Temperature Pour-Over Kettle (OXO, New York City, NY, USA) to 94 °C. The coffee was brewed using a 300-mL glass beaker by adding a constant amount of 200 g of water at 94 °C to an appropriate amount of coffee grounds to yield a desired brew ratio. Water was poured in a circular motion to ensure all the coffee grounds were fully wet and the brew timing was started once the water pouring was completed. The beaker sat on a room temperature wooden countertop with no lid; no additional insulation was provided to mimic typical brewing conditions. At the end of 3, 5, 8, 12, 20, or 30 min into the brew, the whole brew including grounds and coffee were poured into a Hario V60 03 dripper and filter paper (Hario Co., Ltd, Chuo-Ku Tokyo, Japan), where the liquid coffee was separated from the spent grounds using a typical drip brew method with a drip-out time of 1 to 2 min. The mass of the liquid brew was weighed, then a small droplet was pipetted out for TDS measurement. All measurements were performed with three separate trial replicates.

#### Equilibrium in 1-L beaker full immersion brews

The previous two sets of experiments assessed the dynamics of extraction to identify how much time was necessary to achieve equilibrium. That information was then used to guide systematic tests of the impact of brew ratio, brew temperature, and grind size on the equilibrium TDS and extraction. Coffee was ground immediately prior to brewing using a Mahlkönig Guatemala Lab Grinder, typically at grind size setting 5 but also at settings ranging from 2 to 6, corresponding to extra fine to coarse. The median particle sizes are approximately 580, 780, 970, 1160, and 1310 µm at grind size setting 2, 3, 4, 5 and 6 respectively (cf. Supplementary Fig. S1) . The same type of water (Nestlé Pure Life Purified Water) as for the dynamic experiments was heated using an OXO Adjustable Temperature Pour-Over Kettle to a specified temperature between 80 and 99 °C. Coffee was brewed using a 1-L beaker by adding an appropriate amount of water to a constant amount of 30 g of coffee grounds to yield a desired brew ratio. Water was poured in a circular motion to ensure all the coffee grounds were wet. The full immersion brew was left on wooden countertop at room temperature for a target brew time: 60 min for 80 °C brew, 45 min for 85 °C brew, 30 min for 94 °C brew, and 20 min for 99 °C brew, all time values at which the preliminary experiments indicated the TDS of the brew had reached a steady value. The temperature of the brew was measured using an OMEGA HH806AW Wireless Multilogger Thermometer (Omega Engineering, Inc., Norwalk, CT, USA).

After allowing the brew to equilibrate the coffee grounds were filtered out by slowly pouring the brew in a slightly tilted angle through a Hario V60 03 filter paper. The majority of spent coffee grounds remained in the beaker, though a small fraction moved with the brew onto the filter paper. After the brew finished draining through the filter paper, the filter paper and spent grounds on it were carefully returned to the 1-L beaker with the remainder of the spent grounds. The entire beaker, with spent grounds and filter paper, were then placed in an oven for drying. The mass of the filtered brew was weighed, and then a small droplet was pipetted out to measure the TDS. All measurements were performed with three separate trial replicates.

#### Traditional cupping in 12-oz cup full immersion brews

Finally, we performed systematic experiments to assess the strength and extraction of coffees prepared using the traditional cupping method. Coffee was grounded using a Mahlkönig Guatemala 710 Grinder at setting 10 with a median particle size at 548.3 ± 13.4 µm for all five of the coffee blends tested. Particle size distributions were measured by the same laser analyzer (cf. "Dynamics of extraction in 1-L beaker full immersion brews") in triplicate for each coffee blend. Coffee was brewed on the same day of roasting. All coffees were cupped with filtered water containing 180 ppm of dissolved ions, within in the standard range specified by SCA standards. Coffees were brewed individually in a 12-oz glass cup by adding approximately 180 g of 99 °C water to a target amount of coffee grounds, 9 to 14 g, to yield a desired brew ratio. All masses were weighed after adding coffee grounds and adding the water to provide the precise brew ratio. Water was heated using a Bonavita 1.7-L Variable Temperature Electric Kettle (Bonavita World, Woodinville, WA), and was poured in circular motion to ensure all coffee grounds were wet. As per the traditional protocol, four minutes after the brew began a cupping spoon was used to push down and submerge the grinds that had risen to the top layer of the liquid. After that, the brew was left at room temperature for another 10 min; then, a Hario V60 02 filter paper was used to filter out the spent coffee grounds from liquid coffee. After the liquid coffee cooled down to room temperature, a small drop of brew was pipetted out to measure the TDS. All measurements were performed with three separate trial replicates.

#### Quantitative measurements

##### Extraction yield calculation

Brew ratio ${R}_{brew}$ was calculated with experimental mass measurements of fresh coffee grounds and water using Eq. (1). $E$ was calculated from experimental TDS measurements (cf. “Digital refractometer calibration”) and ${R}_{brew}$ using Eq. (17).

##### Oven drying mass measurements

An overview of the experimental procedure involving oven drying method is illustrated in Fig. 1. The 1-L beaker containing spent coffee grounds along with the filtered paper was oven-dried at 100 °C for 24 h. After that, the mass of the whole beaker was measured using a Mettler Toledo PC 2000 Digital Lab Balance (Mettler-Toledo LLC, Columbus, OH, USA). The mass of the dried coffee grounds ${M}_{dried}$ was obtained by subtracting the masses of the beaker and filter paper, each measured prior to coffee brewing. Then, ${E}_{oven}$ was calculated using Eq. (18). The retention ratio ${R}_{ret}$ was calculated as per Eq. (24) from the measured TDS and the mass measurements of water, fresh coffee grounds before brewing, and liquid coffee beverage after filtration.

##### Digital refractometer calibration

The TDS of liquid coffee at room temperature was measured using a digital refractometer (VST, Inc). The refractometer was zeroed with distilled water prior to experimental measurements. Calibration of the digital refractometer was performed using soluble Nescafé Clasico Dark Roast Instant Coffee, by dissolving a specified mass ranging from 0.5 to 4 g of instant coffee in 100 °C deionized water. The mass of instant coffee was measured using a Fisher Scientific XA Analytical (Thermo Fisher Scientific, Waltham, MA, USA). The actual TDS was calculated by mass as.

25 $${TDS}_{calc}=\frac{mass\,\, of \,\,instant\,\, coffee}{mass \,\,of\,\, instant\,\, coffee\,\, + mass\,\, of\,\, water}\times 100\mathrm{\%},$$

and the measured TDS of the solution was measured at room temperature using a VST digital refractometer. The resulting calibration curve (Supplementary Fig. S3) indicates that the refractometer yields excellent measurements of the TDS. All TDS values reported here are those as measured by the refractometer.

### Data analysis

Nonlinear regression of TDS to ${R}_{brew}$ via Eq. (11) was performed using the MATLAB built-in function *lsqcurvefit*, using $K$ as the regression parameter. The corresponding best-fit parameters were evaluated using the built-in function *nlparci* to return a 95% confidence interval. Linear regressions of ${E}_{oven}$ via Eq. (22), TDS calibration, and TDS and E over grind particle size were performed using built-in function *polyfit*. The correlation coefficient (*R*) and p-value of the corresponding linear regressions were calculated using the built-in function *corrcoef*. All plots were generated using MATLAB R2020b (The MathWorks, Inc., Natick, MA).

## Results

### Dynamics of extraction

**Figure 2:**
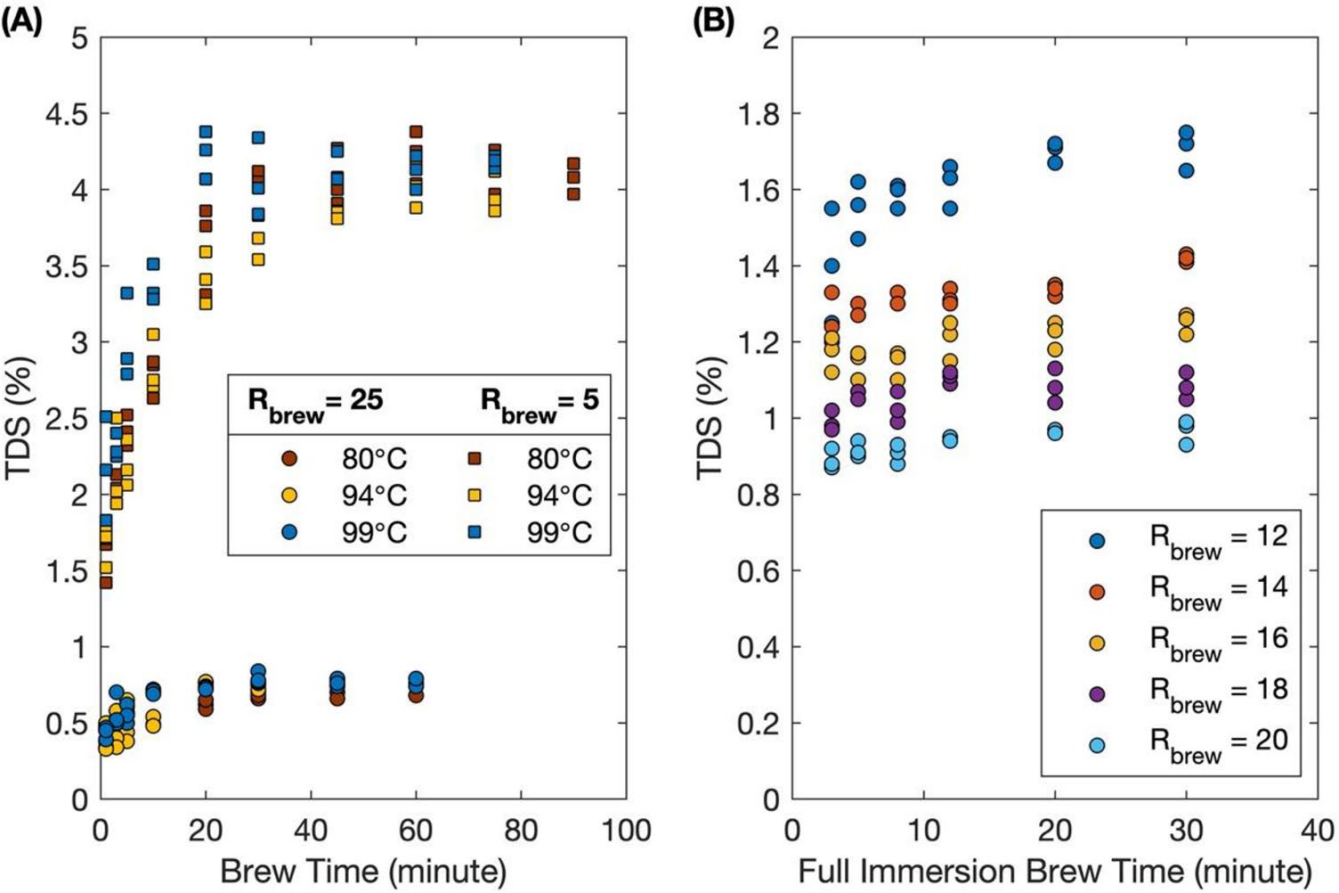
**(A)** Dynamics of extraction for 1-L full immersion brews at two different brew ratios (${R}_{brew}=5$ and ${R}_{brew}=25$) and three different brew temperatures (99 °C, 94 °C, and 80 °C). Scatter plot TDS vs. brew time of full immersion brews with at brew temperatures. Each experiment was performed in triplicate. (**B)** Dynamic TDS vs. full immersion brew time of 300-mL beaker full immersion brews followed by a filtered drip brew at five different brew ratios.

Our dynamic experiments with 1-L brews show that the TDS of full immersion brewed coffee increases with brew time then plateaus after approximately 20 min, for all brew ratios at all brew temperatures tested. For the 1-L full immersion dynamic extractions, coffee brewed with a lower brew ratio yielded higher TDS values, as expected, due to the higher concentrations of soluble species present in the brew, but also took slightly longer to reach equilibrium since the concentration gradient as driving force is smaller between solid and liquid phase compared to the brews with higher brew ratio. For ${R}_{brew}=25$, steady TDS values of approximately 0.7% TDS were obtained in 20 min for all brew temperatures tested. In contrast, for ${R}_{brew}=5$ the brews reached an averaged equilibrium TDS value of approximately 4% at around 45 min into the brew. Similar results were observed with our 300-mL full immersion brews with a single TDS sampling after filtration (Fig. 2b). Brews with ${R}_{brew}$ ranging from 12 to 20 reached steady TDS values ranging from approximately 1.65% to 0.95%, respectively, within about 20 min.

Coffee brewed with higher temperature water reached a steady TDS value more quickly than lower temperature brews did (Fig. 2a). The 99 °C brew with ${R}_{brew}=5$ exceeded 4% TDS within 20 min, while the 94 °C and 80 °C brews took more than 30 min to reach comparable values. Even though the brew temperature did affect the dynamics of full immersion brewed coffee, it did not appreciably affect the final equilibrium TDS. Based our 1-L full immersion brews results, coffee brewed at ${R}_{brew}=5$ had a mean and standard deviation of $4.07\%\pm 0.15\%$ TDS regardless of brew temperature, and TDS was $0.75\%\pm 0.04\%$ for coffee brewed at ${R}_{brew}=25$ over the range of 80 to 99 °C.

### Equilibrium TDS and E in 1-L brews

**Figure 3:**
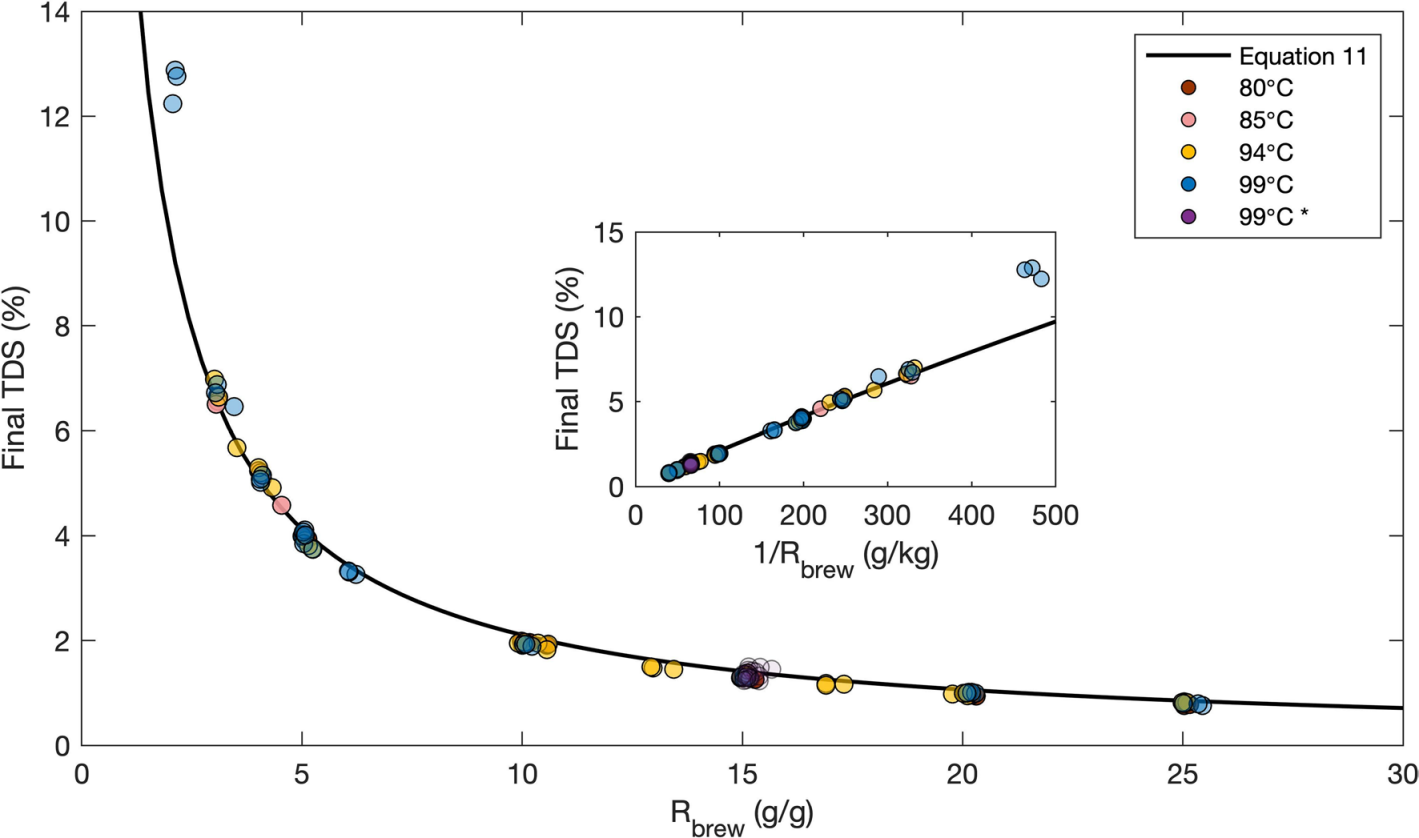
Equilibrium TDS vs. brew ratio for 1-L full immersion brews at various brew temperatures and grind sizes. The asterisk in legend denotes brews with wide range in grind particle sizes (cf. Supplementary Fig. S4). Solid black line is the adsorption–desorption model prediction (Eq. 11), where ${E}_{max}=30\%$, and the equilibrium constant $K=0.717\pm 0.007$ was determined by nonlinear regression with ${R}_{brew}>3$ data points. All experiments were performed in triplicate; a total of 99 distinct TDS values are plotted here. Inset shows the same data and model fit, plotted versus the inverse brew ratio.

After our dynamic tests allowed us to identify conservative over-estimates of the time required to reach equilibrium TDS values, we systematically tested the impact of brew ratio at different brew temperatures and grind sizes to test model prediction Eq. (11). Consistent with the results shown in Fig. 2, we found that increasing the brew ratio strongly decreases the equilibrium TDS. The results are plotted in Fig. 3, which illustrates that the equilibrium TDS is indeed inversely proportional to ${R}_{brew}$, regardless of the brew temperature or grind size over the ranges tested. Our model (cf. Eq. 11) yields an excellent fit versus ${R}_{brew}$ from nonlinear regression, where the best-fit value of equilibrium constant $K$ is $0.717\pm 0.007$ with 95% confidence interval over wide range of brewing conditions. This calculation for $K$ assumes that ${E}_{max}=0.3$ is known with perfect confidence; if we instead treat the quantity ${KE}_{max}$ as a lumped unknown parameter, then regression yields ${KE}_{max}= 0.215\pm 0.002$. The inset in Fig. 3 shows the same TDS data plotted versus $1/{R}_{brew}$ to test the approximate inverse dependence (Eq. 12). The linear fit is excellent, with correlation coefficient R = 0.999 and corresponding p-value $3.66\times {10}^{-105}$, indicating that Eq. (12) provides an accurate approximation.

The most surprising finding is that $K$ did not change appreciably with respect to brew temperature from 80 to 99 °C. The coffee grind particle size did have a very minor effect on TDS. For the brews at ${R}_{brew}=15$ at 99 °C, both the equilibrium TDS and E are negatively correlated with the median of grind particle size with a correlation coefficient of $-0.978$ and $-0.992$ (solid lines in Supplementary Fig. S4), that is, coffee brewed with finer coffee grounds resulted in slightly higher TDS and E. The TDS was $1.36\pm 0.09\%$ over a huge range of grind particle sizes, from a median particle size of 579 µm to 1311 µm (cf. Supplementary Fig. S1), but these small TDS differences are difficult to discern compared to the major effect of ${R}_{brew}$ over the entire range of brew ratios tested.

Equation (11) fit the data extremely well everywhere except at the extreme value of ${R}_{brew}=2$, which had a higher TDS than predicted. Importantly, this brew ratio is less than the average retention ratio of $2.48\pm 0.19$ calculated from Eq. (24), i.e., it is unclear that adding only 2 g of water for every gram of coffee grounds is adequate to fully wet the coffee grounds. Qualitatively, at ${R}_{brew}=2$ the grounds looked like more like a moist sludge than a “fully immersed” brew. Accordingly, we excluded these three data points from the nonlinear regression.

**Figure 4:**
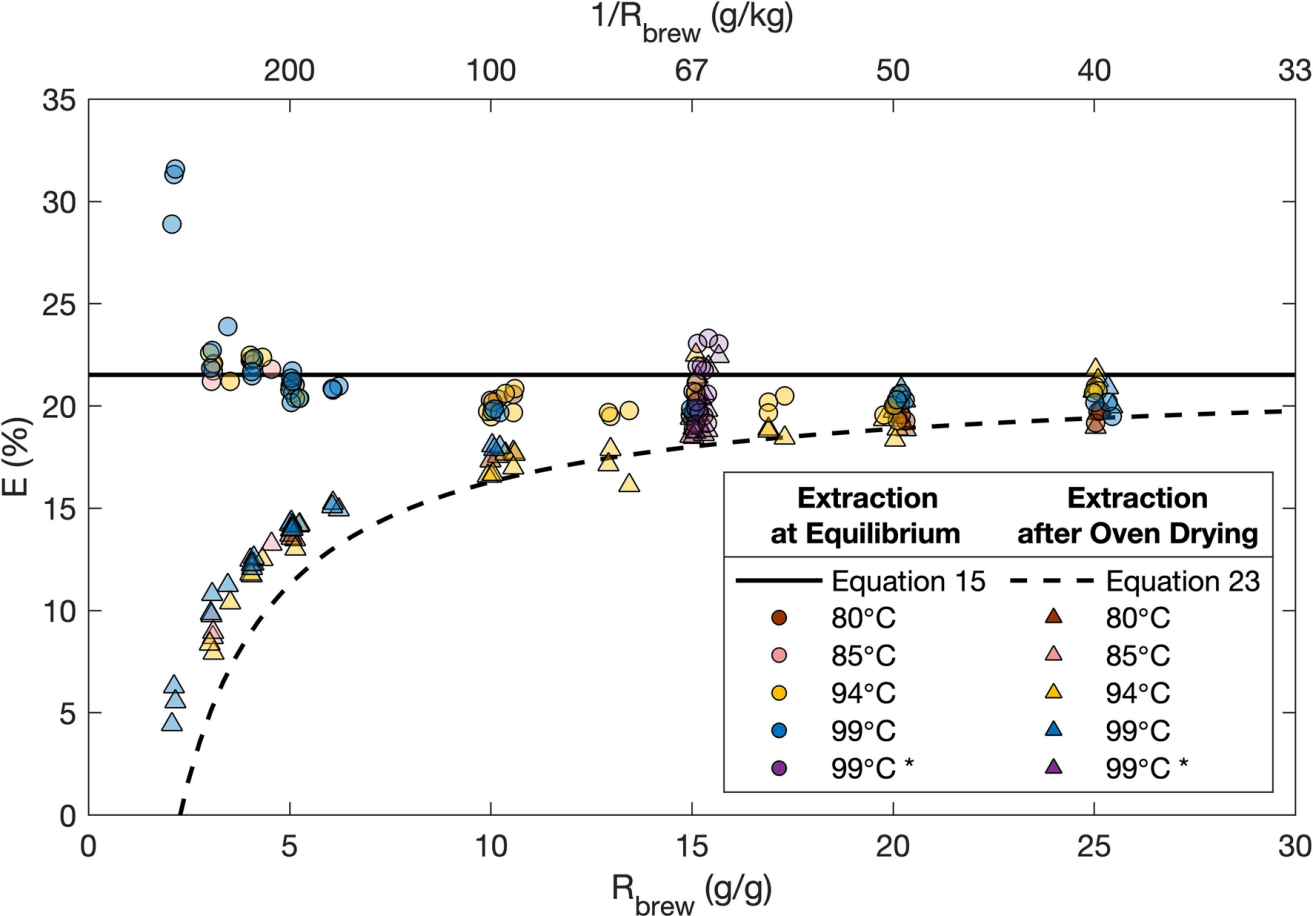
Equilibrium $E$ vs. brew ratio for 1-L full immersion brews at various brew temperatures and grind sizes. The asterisk in legend denotes brews with wide range in grind particle sizes (cf. Supplementary Fig. S4). A secondary x-axis indicates the inverse brew ratio. Solid black line is the adsorption–desorption model prediction for extraction yield (Eq. 15), and the dashed black line is the adsorption–desorption model prediction for oven drying extraction yield (Eq. 23), where ${E}_{max}=30\%$, and the equilibrium constant $K=0.717$. All experiments were performed in triplicate; a total of 99 distinct data points for both $E$ and ${E}_{oven}$ are plotted here.

The corresponding extraction yields for the 1-L brews are shown in Fig. 4. Two different types of extraction measurements are shown here, based on different experimental measurements. First, the “equilibrium extraction” (round data points) are based on the TDS measurements at equilibrium and the known brew ratio, and calculated via Eq. (17). We emphasize that only the TDS values and $${R}_{brew}$$ inform these calculated equilibrium extraction values; no drying data or best fit $$K$$ values are involved. The averaged equilibrium $$E$$ calculated in this manner is $$20.70\pm 1.08\mathrm{\%}$$ for $${R}_{brew}\ge 3$$ (again excluding the ‘moist sludge’ results at $${R}_{brew}=2)$$. This result is in excellent agreement with the theoretical prediction of Eq. (15), $$E =K{E}_{max},$$ which based on the nonlinear regression described in Fig. 3 is $$E =21.5\pm 0.2\%$$ (solid black line, Fig. 4).

The second kind of extraction yield measurements were those obtained by oven drying (triangles in Fig. 4). These ${E}_{oven}$ data points involved only mass measurements of the grounds before brewing and after oven-drying, as per Eq. (18). As expected, ${E}_{oven}$ increased with the brew ratio, then plateaued around 20%, where it approached the values of the equilibrium $E$. This finding is also consistent with the theoretical prediction (dashed black line, cf. Eq. 23), because a much larger fraction of dissolved coffee species was left in the retained coffee in the moist spent grounds; in other words, ${R}_{ret}/{R}_{brew}$ is larger at low ${R}_{brew}$ than in high ${R}_{brew}$ resulting in a smaller value of ${E}_{oven}$. Notably, there appears to be a small but systematic gap between the experimental ${E}_{oven}$ data points and the model prediction curve for ${E}_{oven}$. This effect is presumably due to the exclusion here of the volatilization ratio, which the averaged and standard deviation calculated using Eq. (20) is ${R}_{vol}=0.0234\pm 0.006$. As a comparison, a linear regression of ${E}_{oven}$ via the desorption model prediction (Eq. 22) gives a regressed value of ${R}_{vol}=0.0228$ as the intercept of the best-fit line (Supplementary Fig. S5). In other words, these data suggest that a little more than 2% of coffee solid mass is volatilized during oven 24 h of baking at 100 °C.

Furthermore, we found that the finer the grinds, the higher the observed values of both $E$ and ${E}_{oven}$, at least for a constant brew ratio (Supplementary Fig. S4b). The observed spread, however, was within $4\%$ (i.e., $E$ ranged from about 19 to 23%), over a huge span of coffee particle sizes. Therefore, variation in brew temperature or grind sizes did not considerably alter the extraction yield either for full immersion brewed coffee.

### Equilibrium TDS and E in traditional cupping

**Figure 5:**
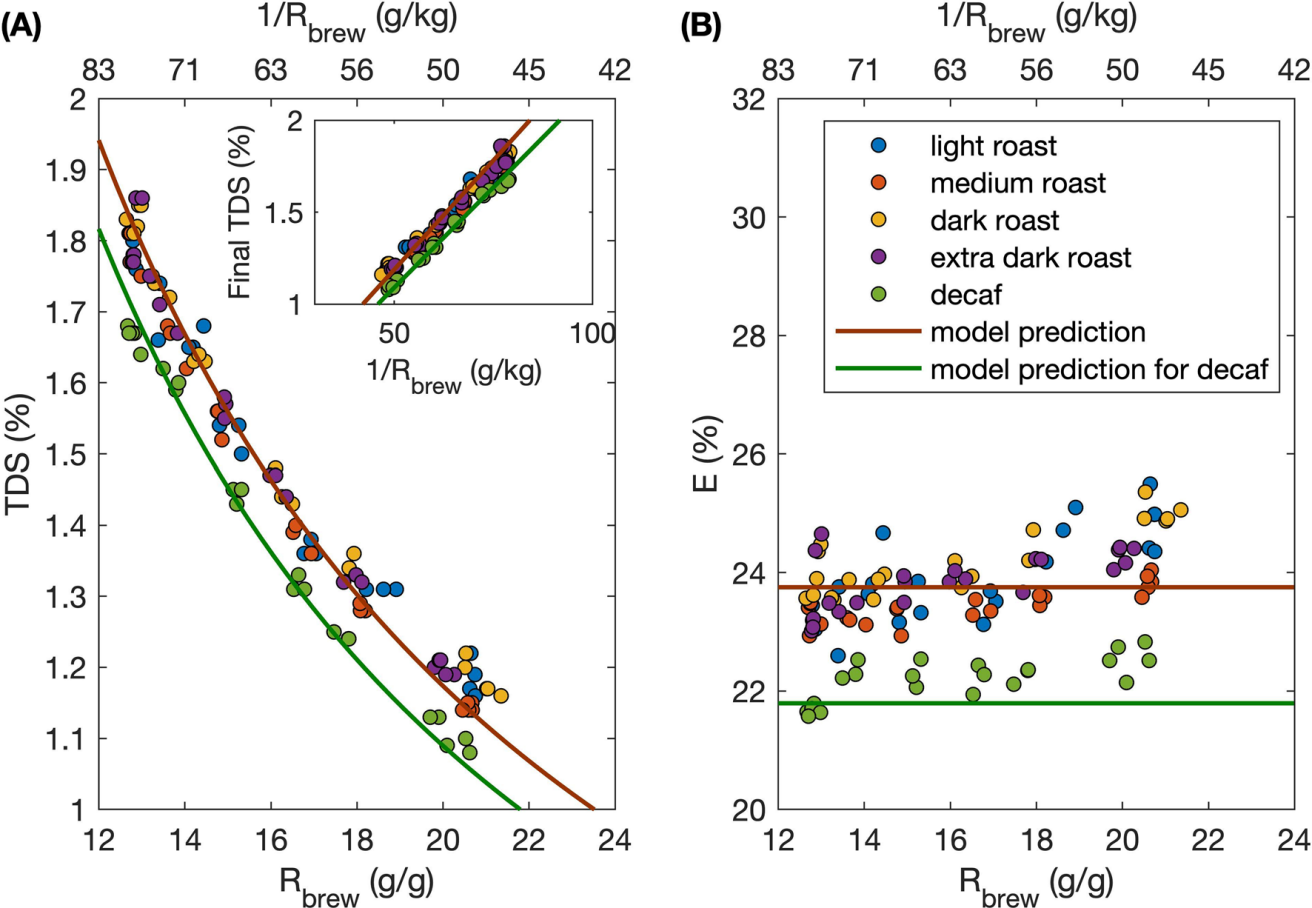
**(A)** Regression of final TDS vs. ${R}_{brew}$ of traditional cupping full immersion brews using different coffee at various roast levels to the adsorption–desorption model (Eq. 11), where ${E}_{max}=30\%$ and the equilibrium constant $K$ serves as the fitting parameter. The best-fit $K$ values with 95% confidence interval are $0.792\pm 0.004$ for caffeinated coffee $0.726\pm 0.006$ for decaffeinated coffee. (**B)** Scatter plot of $E$ vs. ${R}_{brew}$ and model prediction (Eq. 15) for traditional cupping full immersion using different coffee at various roast levels.

Because coffee cupping is one of the most important techniques the coffee industry uses to evaluate coffee quality, full immersion brews following recommended cupping protocol were also investigated systematically versus brew ratio. We again found that the final TDS is inversely proportional to brew ratio for all five different roast levels tested (Fig. 5a). Surprisingly, the four regular coffees tested, ranging from light roast to extra dark roast, had effectively indistinguishable equilibrium TDS values. In contrast, the TDS of the decaffeinated coffee was systematically lower than the regular caffeinated coffee, presumably because it lost some soluble mass when it went through a caffeine extraction process prior to roasting^26^. The regressed equilibrium constants $K$ with 95% confidence interval from our desorption model (cf. Eq. 11) are $0.792\pm 0.004$ for caffeinated coffee and $0.726\pm 0.006$ for decaffeinated coffee, again assuming ${E}_{max}=0.3$ with perfect confidence; the corresponding lumped parameters are $K{E}_{max}=0.240\pm 0.002$ for caffeinated and $K{E}_{max}=0.218\pm 0.002$ for decaffeinated. The inset in Fig. 5a shows the same data plotted versus $1/{R}_{brew}$ to test Eq. (12), and as in Fig. 3 the linear fit is excellent (R = 0.992, p=$6.037\times {10}^{-80}$ and R = 0.997, p = $6.899\times {10}^{-24}$ for caffeinated and decaffeinated coffee, respectively).

Our corresponding measurements of the equilibrium extraction yield (using Eq. 17) again corroborate the model prediction that the extraction yield is insensitive to brew ratio (Fig. 5b). The experimental averaged equilibrium $E$ values with standard deviation are $23.9\pm 0.6\mathrm{\%}$ for caffeinated coffee regardless of roast level and $22.2\pm 0.4\mathrm{\%}$ for decaffeinated coffee, which accord with our model prediction of $23.79\%$ and $21.79\%$ for caffeinated and decaffeinated coffee, respectively (solid lines, Fig. 5b).

## Discussion and conclusions

The overarching conclusion from this work is that full immersion coffee brewing is well characterized by an equilibrium desorption model that assumes not all of the soluble material in the coffee grounds will go into solution. The experimental measurements of TDS and $E$ over a wide range of conditions strongly accord with the model predictions, indicating that use of a single averaged equilibrium constant suffices to capture the overall behavior of the complicated mixture of individual chemical components.

### Insensitivity to temperature

A surprising aspect of our results is the pronounced insensitivity to the brew temperature. Although the dynamics of extraction were strongly affected by the brew temperature, with hotter temperatures yielding an equilibrium TDS more quickly than lower temperatures (Fig. 2a), the final value of the TDS and corresponding $E$ were insensitive to the brew temperature over the wide range 80 °C to 99 °C. We emphasize, however, that this result does not mean that the brews prepared at these different temperatures will necessarily taste the same. The sensory impressions perceived by individuals upon tasting the brew depend on the concentration of individual chemical species within it, not the overall mass fraction of dissolved solids; it is possible that two brews could have the same TDS and $E$ but nonetheless have different underlying chemical compositions and hence different sensory profiles. Furthermore, measurements of the TDS do not reflect the presence of emulsified oil droplets or non-dissolved colloids, both of which affect the sensory profile and conceivably could vary more dramatically with brew temperature.

Despite these caveats, however, there is reason to suspect that the brew temperature might not appreciably alter the final sensory profile full immersion brews. Work by Batali et al. demonstrated that drip brew coffees prepared to identical TDS and $E$ values but using different brew temperatures had sensory profiles that were indistinguishable to trained panelists^27^. Although those drip brew experiments were performed over a narrower temperature range (87 °C to 93 °C), they raise the possibility that a similar lack of sensory profile dependence on brew temperature might occur with full immersion brews over a wider range of temperatures. Detailed sensory descriptive experiments must be performed with full immersion brews to test this hypothesis. Furthermore, the tests reported here did not examine “cold brews,” which are traditionally performed either at room temperature (near 24 °C) or refrigerator temperature (near 4 °C). Additional experiments are necessary to examine the behavior of full immersion cold brew.

### Strength and extraction control via brew ratio

Although the brew temperature over traditional hot brew temperature ranges might not affect the final brew appreciably, our results clearly demonstrate that the brew ratio has a huge influence: our model and experimental results both indicate that the TDS varies inversely with ${R}_{brew}$. Because the sensory profile is strongly correlated with the TDS, this result indicates that the simplest way to modify the sensory profile for full immersion brewing is to modify the brew ratio. Changes in the brew temperature, grind size, and agitation will alter the dynamics of the extraction, but not the composition of the final brew itself; in contrast, the brew ratio directly controls the final composition.

In contrast to the ease with which TDS is varied, our results indicate that full immersion brewing offers little control over the extraction yield. To good approximation over typical brew ratios, the E is predicted to be independent of the brew ratio (cf. Eq. 15). Specifically, based on our measurements, it appears that full immersion brews at equilibrium invariably yield an $E$ near 21%, and modifications in the brew temperature, grind size, or brew ratio do not alter the final E appreciably. We emphasize that this “equilibrium $E$” is what matters for the beverage because it directly corresponds to the beverage that is to be consumed, whereas the "effective $E$" measured via oven drying is potentially misleading because it includes dissolved but retained mass in the filtered spent coffee grounds and thus provides a significant underestimate of the actual $E$ in the beverage.

Another notable observation is that the traditional cupping brews had a slightly higher $K$ and $E$ than the 1-L beaker equilibrium brews. The reason for this difference is unclear. One possible confounding factor is that the coffees for the traditional cupping tests were brewed the same day that they were roasted, without a lengthy degassing period as allowed for the 1-L tests reported here. The release of CO_2_ and other volatile compounds happens with a varied degassing speed depending on the roast level and roast speed, and can cause mass losses on the order of 10 mg/g of roasted coffee, equivalent to a 1% mass loss^28^. If, however, these volatile species were still present in the roasted beans when weighed, but then they readily escape into the gas phase upon brewing, then the extraction yield should be lower, rather than higher as observed. Another possible factor is that slow chemical reactions continue to occur after roasting^29^; if these reactions alter the solubility or equilibrium desorption coefficients, then the average equilibrium TDS and extraction yield will be affected accordingly. More specific tests will be necessary to test this hypothesis.

### Implication for practitioners

In terms of practical implications, the inability to alter the equilibrium $E$ in full immersion brews effectively removes a “knob” available to fine tune the desired sensory profile. Prior work with drip brews has shown that brews prepared at the same TDS but different $E$ have significantly different sensory profiles^9^. In terms of the classic Coffee Brewing Control Chart, full immersion brews at equilibrium only allow a brewer to move vertically with respect to TDS (by changing the brew ratio), but not horizontally with respect to $E$. This observation suggests the hypothesis that full immersion techniques cannot easily produce brews with maximum amounts of sourness and citrus (as observed with high TDS, low $E$) or maximal amounts of sweetness (as observed at low TDS, low $E$) or maximal amounts of tea/floral (as observed at low TDS, high E). Lower $E$ values can be obtained in full immersion only by cutting the brew short (i.e., not allowing it to reach a final steady TDS value); higher $E$ values cannot be obtained with a single full immersion brew. In contrast, flow brewing methods like drip brew provide more flexibility, since the flow rate, brew ratio, and total brew time can be varied to achieve desired combinations of TDS and $E$. This increased flexibility, however, is potentially offset by the increased expense and complexity of drip brew equipment; cupping for example is widely preferred by coffee industry professionals because of its simplicity. Coffee brewers should take this tradeoff between flexibility and simplicity into account when choosing between flow based and full immersion brewing methods.

## Supplementary Information

Supplementary Information.
