# Supplementary material for: An equilibrium desorption model for the strength and extraction yield of full immersion brewed coffee
Source: Sci Rep. 2021 Mar 25;11:6904. doi: 10.1038/s41598-021-85787-1 (PMC7994670; doi:10.1038/s41598-021-85787-1)
Supplement: Supplementary file 1 — Supplementary Information. [file 41598_2021_85787_MOESM1_ESM.pdf]

An Equilibrium Desorption Model for the Strength and Extraction Yield of Full Immersion Brewed Coffee

Liang, Jiexin[1], Chan, Ka Chun[2], and Ristenpart, William D.[2*]

Author affiliations
[1]Department of Food Science and Technology
University of California, Davis
One Shields Avenue
Davis, CA, 95616 USA

[2]Department of Chemical Engineering
University of California, Davis
One Shields Avenue
Davis, CA, 95616 USA

***Corresponding author**
wdristenpart@ucdavis.edu

Supplementary Figures:

S1. Particle grind size distributions.

S2. Final brew temperature vs. brew ratio.

S3. Calibration curve for TDS via refractometry.

S4. Equilibrium TDS and E vs. particle grind size.

S5. Oven extraction measurement (E_{oven}) versus model prediction.

Supplementary Tables:

S1. Table of nomenclature.

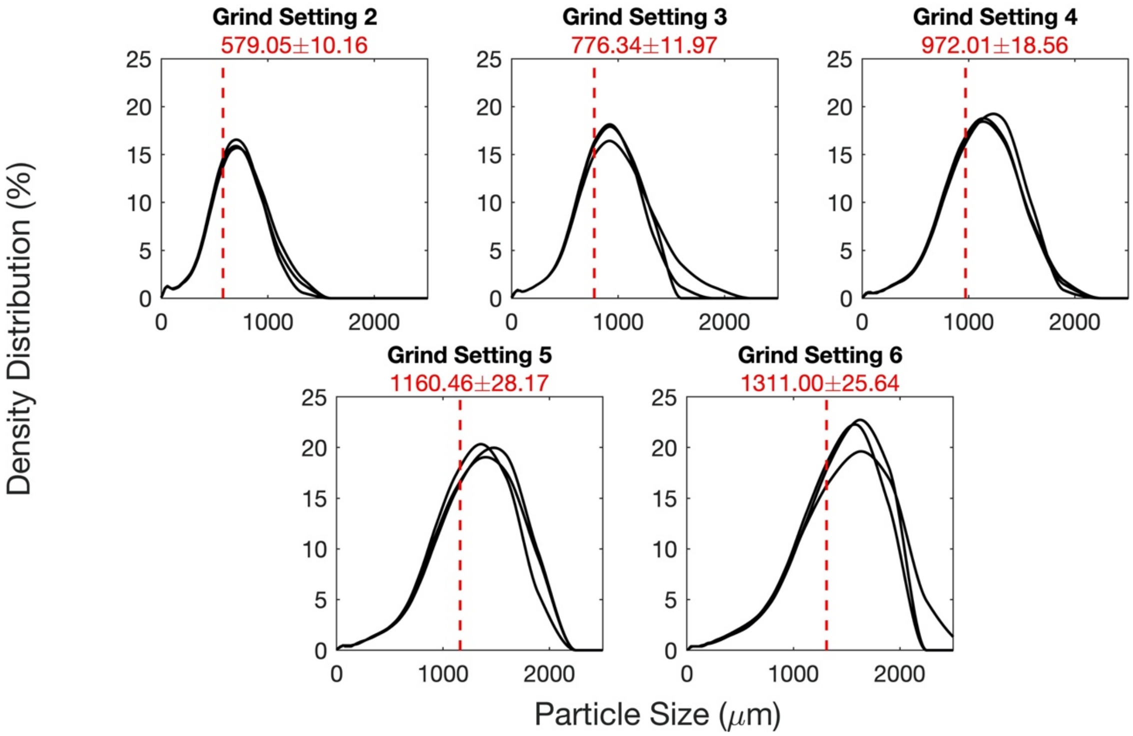

Supplementary Figure S1. Particle size distribution at five different grind size settings for the Mahlkönig Guatemala Lab Grinder, each measured in triplicate. Distributions are expressed in terms of the normalized differential density distributions of the given particle size. The median particle size, x_{50}, with standard deviation at each grind size setting is indicated by red dashed line.

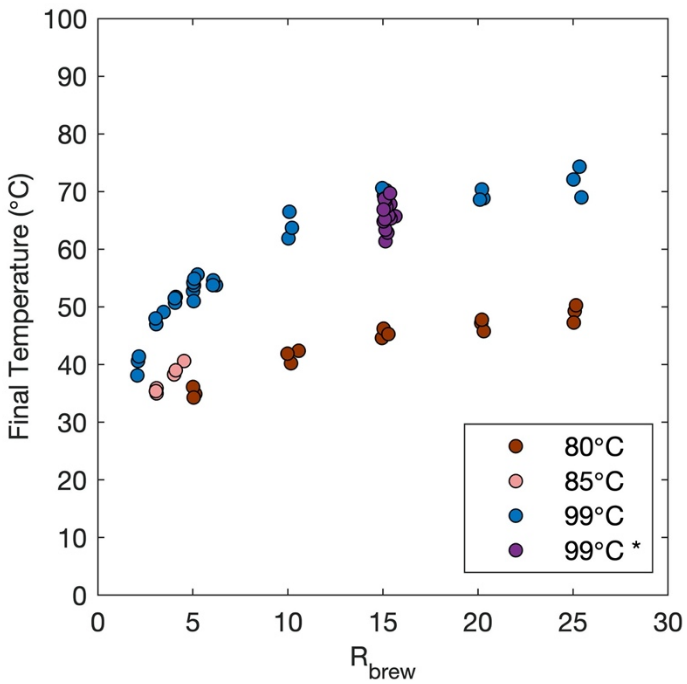

Supplementary Figure S2. Final temperature vs. R_{brew} of 1-L full immersion brews at various brew temperature and grind sizes.

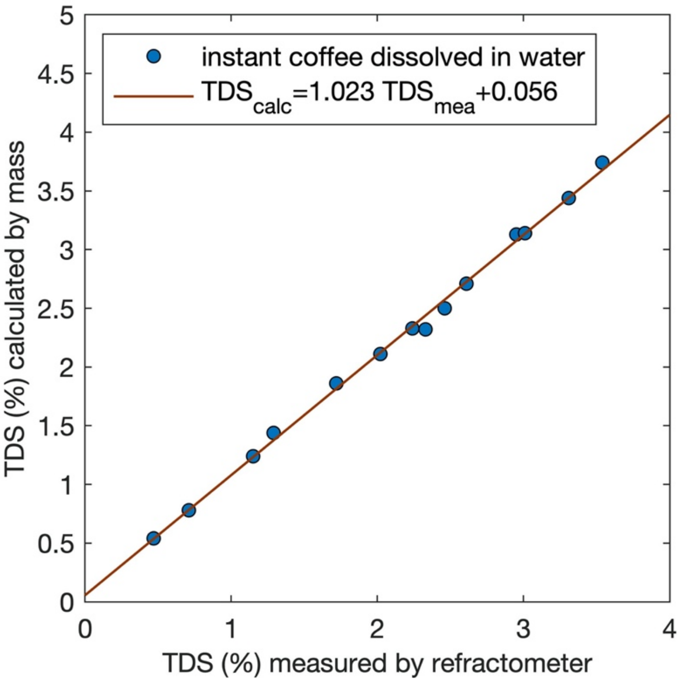

Supplementary Figure S3. Calibration of TDS measurement from VST Digital Refractometer by linear regression from the calculated TDS value by mass TDS_{calc} using Eq. 25 and the measured TDS value TDS_{mea} of dissolved pure instant coffee in boiling water. The correlation coefficient between TDS_{mea} and TDS_{calc} is 0.999, and the p-value is 8.818×10^{-17}.

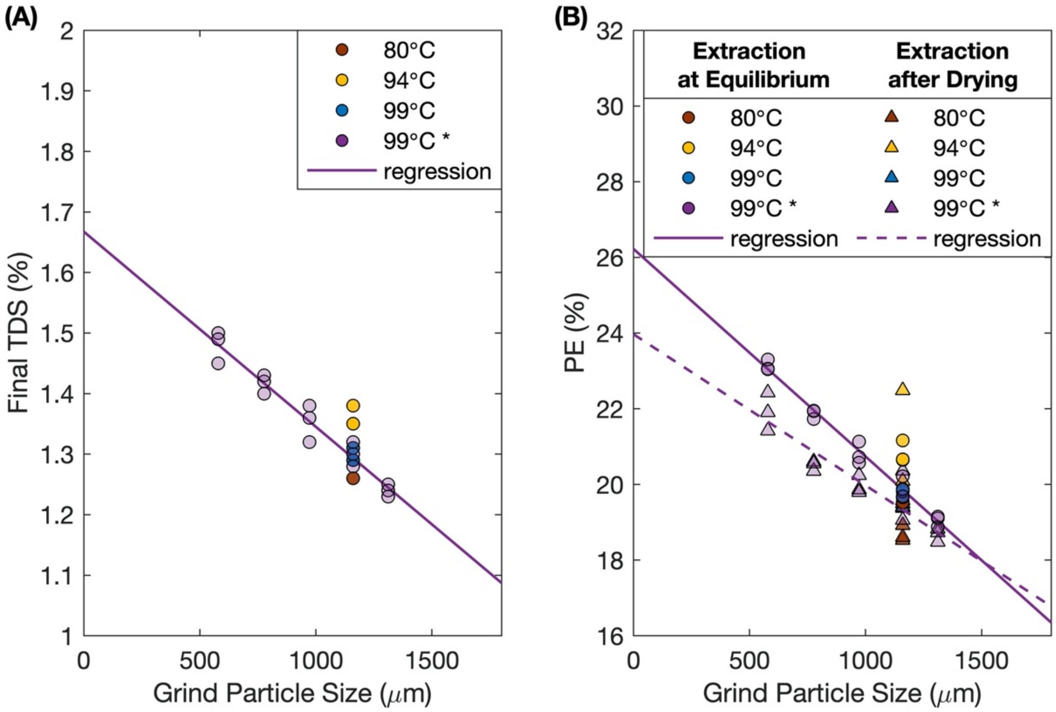

Supplementary Figure S4. (A) Equilibrium TDS vs. median particle grind size, x_{50}, (cf. Supplementary Fig. S1) for 1-L full immersion brews at $R_{brew} = 15$. The solid line denotes the linear regression $TDS = -0.0003x_{50} + 1.67$, for the brews at 99°C, with R=-0.978, p= 2.40×10^{-12}. (**B)** Equilibrium E vs. median particle grind size, for 1-L full immersion brews at $R_{brew} = 15$. The solid and dashed lines indicated linear regressions for E and E_{oven}, respectively, at 99°C, with corresponding equations $E = -0.0055x_{50} + 26.2$ and $E_{oven} = -0.0039x_{50} + 23.9$, The corresponding regression statistics are, respectively, R=-0.992, p= $7.21 \times 10^{-16}$and R=-0.933, p= 1.65×10^{-8}.

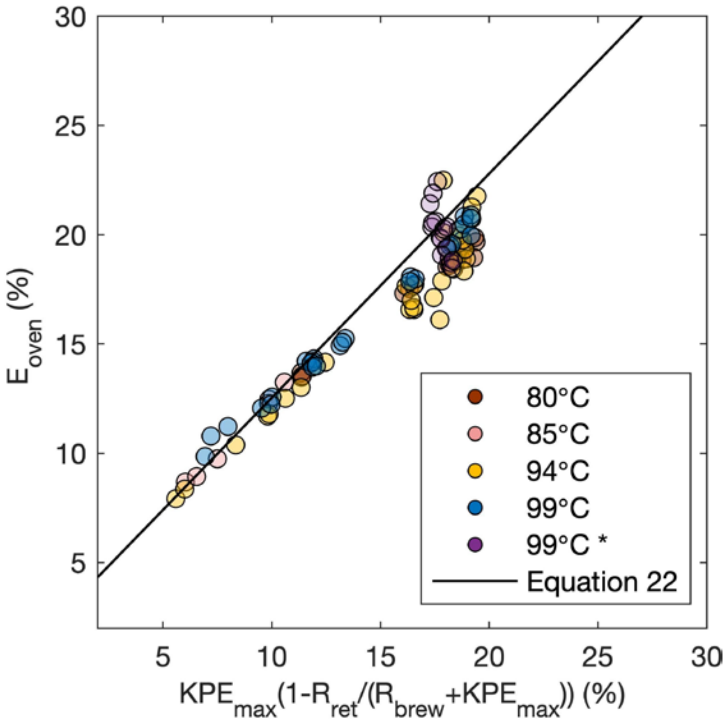

Supplementary Figure S5. Equilibrium E_{oven} vs. $KE_{max}\left(1-\frac{R_{ret}}{R_{brew}+KE_{max}}\right)$ of 1-L full immersion brews at various brew temperatures and grind sizes. The asterisk in legend denotes brews with wide range in grind particle sizes, cf. Supplementary Fig. S4. Solid black line is the adsorption-desorption model prediction for Equilibrium E_{oven} (Eq. 22) with $E_{max}=30\%$, and the volatization ratio $R_{vol}=0.0228$ was determined by the intercept of this best-fit line with slope $=1.03$ through linear regression (R=0.997, p=1.10×10^{-38}). As a comparison, the averaged volatilization ratio with standard deviation calculated from Eq. 20 using measurable quantities is $R_{vol}=0.0234\pm0.006$.

Symbol	Units	Definition
C_A	-	mass concentration of adsorbed soluble coffee species
C_D	-	mass concentration of dissolved soluble coffee species (TDS)
C_{tot}	-	total concentration of extractable coffee species
E	-	extraction yield at equilibrium
E_{max}	-	maximum possible extraction yield of coffee grounds
E_{oven}	-	measurable extraction yield using the oven-drying method
K	-	species-averaged equilibrium constant
k_A	$1/\mathrm{second}$	first order rate constant for adsorption of soluble species
k_D	$1/\mathrm{second}$	first order rate constant for desorption of soluble species
M_A	grams	mass of adsorbed coffee species
M_{brew}	grams	mass of liquid beverage that could be consumed
M_D	gram	mass of dissolved coffee species
M_{dried}	grams	mass of dried spent grounds after oven drying
M_g	grams	Initial mass of coffee grounds added to the brewer
M_L	grams	mass of liquid present in the brew after dissolution of soluble species
M_{ret}	grams	mass of retained liquid coffee within the moist spent coffee grounds
M_{spent}	grams	mass of spent moist coffee grounds
M_{vol}	grams	mass of volatile coffee solids lost to the gas phase during oven drying
M_w	grams	Initial mass of water added to the brewer
R_{brew}	-	water to coffee mass brew ratio
R_{ret}	-	retention mass ratio (retained liquid per mass coffee grounds)
R_{vol}	-	volatilization mass ratio (evaporated solids per mass coffee grounds)
TDS	-	total dissolved solids (mass dissolved solids per mass of liquid)
x_{50}	μm	median of particle grind size distribution

Supplementary Table S1. Table of nomenclature. A hyphen under "Units" denotes a dimensionless quantity.
